# Neural Plasticity after Congenital Brain Lesions

**DOI:** 10.1155/2019/9154282

**Published:** 2019-05-02

**Authors:** Simona Fiori, Martin Staudt, Roslyn N. Boyd, Andrea Guzzetta

**Affiliations:** ^1^Department of Developmental Neuroscience, IRCCS Stella Maris Foundation, Pisa, Italy; ^2^University Children's Hospital, Department of Pediatric Neurology and Developmental Medicine, University of Tübingen, Germany; ^3^Clinic for Neuropediatrics and Neurorehabilitation, Epilepsy Centre for Children and Adolescents, Schön Klinik Vogtareuth, Germany; ^4^Queensland Cerebral Palsy and Rehabilitation Research Centre, UQ Child Health Research Centre, Faculty of Medicine, The University of Queensland, South Brisbane, Queensland, Australia; ^5^Department of Clinical and Experimental Medicine, University of Pisa, Pisa, Italy

In the past decades, interest has grown on the mechanisms of early brain plasticity and their implications for newborns and infants with brain damage. Early brain damage triggers complex processes of adaptive neuroplasticity, which involve various functional systems and are highly influenced by the environment. Understanding the complex process of reorganization of neural functions through adaptive plasticity is a fast-growing field of research that has the potential to prompt more targeted and evidence-based interventions to promote neurodevelopment. The objective of this special issue was to collect scientific reports and literature reviews, from both animal models and human studies, contributing to a better understanding of the characteristics of early adaptive neuroplasticity following early brain damage.

Understanding in vivo mechanisms of damage and plasticity needs animal models. Unilateral brain damage is probably the most studied model for the characterization of brain plasticity. The review by M. Gennaro et al. is aimed at exploring developmental ischemic stroke pathophysiological mechanisms, focusing on key factors contributing to neonatal brain vulnerability and summarizing current available stroke models in animal labs. These models are highly informative to human studies, as they identify mechanisms of neuroplasticity in controlled experimental conditions often using invasive means of investigation. Fortunately, noninvasive neuroimaging and electrophysiological techniques are now widely available and make it possible to describe the specificity of early mechanisms also in humans.

Evidence of early plasticity was found, for example, by C. Simon-Martinez et al. who explored determinants of impaired upper limb sensory and motor functions and corticospinal tract wiring by using transcranial magnetic stimulation and related this to lesion topography as assessed by a visual semiquantitative scale for brain lesion severity on MRI in CP. In an innovative ultra-high-field MRI technique, S. Fiori et al. explored the plasticity of sensory systems in young adults with congenital brain lesions. Their findings support the crucial role of topography of brain damage and reorganized somatosensory areas in relation to deficits of hand sensory and possibly motor function. In another study, S. S. Geertsen et al. explored the electrophysiological mechanisms of coactivation of muscles and high step-to-step variability of gait in adults with early brain lesion and CP, which are the result of a long learning process involving predictive coding of the sensory consequences of movement.

Indeed, different factors interact with brain adaptive plasticity potentials, influencing the natural history of cerebral palsy in the first years. To understand these mechanisms, K. Klingels et al. collected longitudinal functional data over a 5-year time period in children with unilateral CP. They observed increasing limitations in the passive range of motion and improvement in capacity measures, while the spontaneous use of the impaired limb in bimanual tasks became less effective after the age of 9 years.

Evolution of upper limb function in children with unilateral CP and the factors that influence these time trends can provide guidance in delineating treatment priorities. The effects of new treatment options have recently been the object of growing interest. For example, E. Inguaggiato et al. found an increase in unilateral hemiparetic hand function (manual dexterity) in children and young adults with unilateral CP, with improvements emerging immediately at the end of transcranial direct current stimulation (tDCS) and persisting for at least 90 minutes. The action observation network (AON) was studied by G. Sgandurra et al. in children with unilateral CP. They demonstrated similarities compared to AON in typically developing children and propose an AON paradigm to explain and predict the efficacy of rehabilitation in unilateral CP children and to investigate the effects of plasticity induced by specific rehabilitation programs. They also demonstrated that a more lateralized AON corresponded to a worse impaired hand performance, as an example of maladaptive plasticity. In another paper, T. L. Rich et al. applied transcranial direct current stimulation (tDCS) as an opportunity to open a window for plasticity in unilateral CP, showing that a single application of anodal tDCS over the affected M1 can improve, in a safe and feasible way, possible but inconsistent gains in hand function.

Of course, neural plasticity has not only implications in the organization of motor and somatosensory function. Visual deficits, for example, are increasingly recognized as being part of the clinical picture in most cases. Confirmation comes from the paper of G. Ickx et al. who explored visuospatial attention in unilateral CP and demonstrated clinical differences according to the laterality of brain and lesion timing: children with corticosubcortical lesions more frequently presented visuospatial attention deficits than children with periventricular brain lesion, which might be the results of impacted function or plasticity mechanisms.

